# Therapeutic implications of statins in heart failure with reduced ejection fraction and heart failure with preserved ejection fraction: a review of current literature

**DOI:** 10.12688/f1000research.28254.1

**Published:** 2021-01-12

**Authors:** Chol Techorueangwiwat, Chanavuth Kanitsoraphan, Panupong Hansrivijit

**Affiliations:** 1 University of Hawaii Internal Medicine Residency Program, Honolulu, HI, 96813, USA; 2 Department of Internal Medicine, UPMC Pinnacle, Harrisburg, PA, 17011, USA

**Keywords:** statins, heart failure, mortality, HFrEF, HFpEF, prevention, HMG-CoA inhibitor

## Abstract

Statins are one of the standard treatments to prevent cardiovascular events such as coronary artery disease and heart failure (HF). However, data on the use of statins to improve clinical outcomes in patients with established HF remains controversial. We summarized available clinical studies which investigated the effects of statins on clinical outcomes in patients with HF with reduced ejection fraction (HFrEF) and HF with preserved ejection fraction (HFpEF). Statins possess many pleiotropic effects in addition to lipid-lowering properties that positively affect the pathophysiology of HF. In HFrEF, data from two large randomized placebo-controlled trials did not show benefits of statins on mortality of patients with HFrEF. However, more recent prospective cohort studies and meta-analyses have shown decreased risk of mortality as well as cardiovascular hospitalization with statins treatment. In HFpEF, most prospective and retrospective cohort studies as well as meta analyses have consistently reported positive effects of statins, including reducing mortality and improving other clinical outcomes. Current evidence also suggests better outcomes with lipophilic statins in patients with HF. In summary, statins might be effective in improving survival and other clinical outcomes in patients with HF, especially for patients with HFpEF. Lipophilic statins might also be more beneficial for HF patients. Based on current evidence, statins did not cause harm and should be continued in HF patients who are already taking the medication. Further randomized controlled trials are needed to clarify the benefits of statins in HF patients.

## Introduction

Statins are blood-cholesterol-lowering drugs that are used worldwide to prevent cardiovascular diseases. They inhibit the 3-hydroxy-3-methylglutaryl coenzyme A (HMG-CoA) enzyme and thereby reduce the synthesis of cholesterol in the liver ^1^. Statins also upregulate the expression of low-density lipoprotein (LDL) receptors on the cell membrane ^2^. Clinically, statins can reduce LDL by 20–60% and triglyceride by 10-40%, and increase high-density lipoprotein (HDL) by 5%-15% ^3– 5^. Statins have a dramatic effect on reducing cardiovascular events and the data from one clinical trial has shown that the risk for major vascular events is reduced by 22% for each 1 mmol/L reduction in LDL ^6^.

The role of statins in the primary and secondary prevention of coronary artery disease is well established ^7– 9^, irrespective of patients’ cholesterol level ^10^. Robust evidence also exists supporting the role of statins in the prevention of new incident heart failure (HF) ^11^. Given their many pleiotropic effects, statins were hypothesized to also be effective in improving outcomes in patients with established HF ^12^. However, the evidence for the use of statins in patients with HF is controversial ^13– 17^, so the role of statins in this population remains unclear. Current guidelines have not recommended the routine use of statins in most patients with HF without other indications for their use (e.g. coronary artery disease, hypercholesterolemia), but suggest that statins can be continued for patients who were already on treatment ^18^. More recent evidence is emerging suggesting potential benefits of statins in patients with HF to improve clinical outcomes ^19– 22^.

In this review, we summarized potential mechanisms of statins that might be beneficial for patients with HF and available clinical studies which investigated the effects of statins on clinical outcomes in patients with HF with reduced ejection fraction (HFrEF) and HF with preserved ejection fraction (HFpEF).

## Potential mechanisms of statins in heart failure

Multiple mechanisms have been described to explain the potential benefits, as well as harms, of statins in HF. Various experimental and clinical studies have described pleiotropic effects of statins independent of their lipid-lowering ability that might improve the outcomes in patients with HF ^23, 24^. Statins stabilize atheromatous plaques and possess antiatherogenic properties that help reduce atheroma volume and prevent formation of new atherosclerotic lesions, which in turn reduces ischemic burden and results in less cardiac tissue damage ^11, 25^. Statins promote left ventricular healing after myocardial infarction by improving left ventricular remodeling ^26^ and modulating endothelial function ^27^. Statins have also been found to possess anti-inflammatory properties ^28– 30^ leading to the reduction of oxidative stress in vascular and myocardial tissues ^31, 32^. Other important mechanisms of statins have been reported, including enhancement of endothelial function ^33^, inhibition of the action of angiotensin II ^34^, reduction of sympathetic activities ^35^, delaying apoptosis ^32^, and suppression of arrhythmogenesis ^36^. In addition, statins may exert their pleiotropic effects through epigenetic modifications as they can act as histone deacetylase inhibitors ^37, 38^. Despite two large randomized controlled trials (RCTs) exploring the effects of statins in systolic HF showing that statins did not improve all-cause mortality or other major cardiovascular outcomes ^13, 14^, some meta-analyses and observational studies have shown the contrary ^15, 20, 22, 39^. This is discussed in detail below for HFrEF and HFpEF, respectively.

There are two types of statins: hydrophilic (e.g. rosuvastatin, pravastatin) and lipophilic (e.g. atorvastatin, simvastatin). Each type has different properties. Hydrophilic statins employ active transport into hepatocytes, whereas lipophilic statins enter cells by passive diffusion and therefore are more easily absorbed by peripheral tissues, including myocardial cells ^40– 42^. Current evidence suggest that the benefits of statins also depend on their lipophilicity, thus the effects in populations with HF should not be expected to be identical across all statin drug class ^41, 43, 44^.

Multiple mechanisms have also been proposed that raise concerns for the adverse effects of statins in patients with HF. Low serum total cholesterol has been associated with higher mortality in patients with HF for instance ^45– 50^; however, it is unclear if low cholesterol is an independent risk factor for poor outcomes or is merely a surrogate marker for cardiac cachexia in patients with more severe disease ^51^. It has also been hypothesized that hepatic and intestinal congestion in patients with HF can impair hepatic cholesterol synthesis and cholesterol absorption, respectively, therefore resulting in lower plasma cholesterol level ^46, 52^. In a South Korean cohort study analyzing 2,797 patients with HF, those with lower serum total cholesterol had more markers of severe diseases (e.g. lower blood pressure, lower serum sodium), but low cholesterol itself did not affect the clinical outcomes in a propensity score matched analysis ^53^. Another mechanism through which statins might adversely affect HF is that statins have been shown to reduce serum coenzyme Q10 level, an important enzyme in myocardial bioenergetics ^54^. Studies have reported that low serum coenzyme Q10 level was associated with increased mortality in patients with HF ^55, 56^. Nevertheless, two large randomized trials have shown that statin use was not associated with significant worsening of clinical outcomes or adverse events in HF populations ^13, 14^.

In this non-systematic review, we searched Ovid MEDLINE for randomized controlled trials on the use of statins in heart failure patients. The review covers from current clinical evidence to pathophysiology review accrued from bench research. The search terms used are depicted in Table 1.

**Table 1.** Search strategies on OVID Medline.

No.	Search term(s)
1	Statins OR statin
2	Heart failure OR CHF OR cardiomegaly
3	HFpEF OR diastolic heart failure
4	HFrEF OR systolic heart failure
5	2 OR 3 OR 4
6	1 AND 5
7	Filters applied: human, clinical study, English language

## Statins in heart failure with reduced ejection fraction: current evidence

A list of relevant clinical studies on the use of statins in HFpEF patients is presented in Table 2. Of these studies, the best evidence to date comes from two large randomized placebo-controlled trials: CORONA ^14^ and GISSI-HF ^13^. Neither of these trials found benefits of statin therapy in patients with HF. The CORONA trial recruited 5,011 patients with ischemic HFrEF (mean age 73 years and mean left ventricular ejection fraction [LVEF] 31%) and randomized the subjects to either rosuvastatin 10 mg daily or a placebo. After the median follow-up of 32.8 months, there was no significant reduction in the primary endpoints, including the composite of death from cardiovascular causes, nonfatal myocardial infarction, and nonfatal stroke, and no significant difference between groups ^14^. However, a post-hoc analysis of this trial showed that, when comparing outcomes by N-terminal pro-B-type natriuretic peptide (NT-proBNP) tertile, patients in the lowest NT-proBNP tertile (< 103 pmol/L) had a significantly lower risk of the primary outcomes ^57^. In addition, rosuvastatin also reduced the incidence of hospitalization for cardiovascular causes which has also been found by other researchers ^14, 58^. Secondly, the GISSI-HF trial randomized 4,574 patients with HF from any cause (mean age 68 years, mean LVEF 33%) to receive either 10 mg daily of rosuvastatin or a placebo. Similar to the result of the CORONA trial, there were no significant differences in the co-primary outcomes of all-cause mortality or in the combined endpoints of death or hospitalization for cardiovascular causes ^13^.

**Table 2.** Relevant clinical studies with the primary objective of determining the effects of statins in patients with heart failure with reduced ejection fraction.

Study, year	Study design	N	Type of statin	Follow up time	Primary endpoints	Results
Horwich *et al.* 2004 ^59^	Prospective cohort	551	N/A	12 months	All-cause mortality	Reduction in all-cause mortality (HR 0.41; 95% CI 0.18 to 0.94)
Sola *et al.* 2005 ^60^	Prospective cohort	446	Atorvastatin Fluvastatin Pitavastatin Simvastatin	24 months	All-cause mortality and hospitalization for heart failure	Reduction in all-cause mortality (HR 0.71; 95% CI 0.57–0.84; P < .001) and hospitalization for heart failure (HR 0.77; 95% CI 0.62–0.91; P < .001).
Hong *et al.* 2005 ^61^	Prospective cohort	202	Simvastatin	12 months	N/A	Reduction in mortality, restenosis rate, and repeat PCI among patients with ischemic systolic HF who underwent PCI for acute MI
Go *et al.* 2006 ^62^	Prospective cohort	24, 598	N/A	2.4 years	All-cause mortality and hospitalization for heart failure	Reduction in all-cause mortality (adjusted HR 0.76; 95% CI 0.72-0.80) and hospitalization for heart failure (adjusted HR: 0.79; 95% CI; 0.74-0.85)
Kjekshus *et al.* 2007 (CORONA trial) ^14^	RCT	5,011	Rosuvastatin	32.8 months	Composite of CV death, nonfatal MI, and nonfatal stroke,	No significant reduction in primary outcome (HR 0.92; 95% CI 0.83 to 1.02; P=0.12)
Huan *et al.* 2007 ^63^	Prospective cohort	900	N/A	36 months	All-cause mortality	Better survival in patients who were on statins
Coleman *et al.* 2008 ^64^	Prospective cohort	1204	N/A	31 months	All-cause mortality, VT/VF	Reduction in all-cause mortality (adjusted HR 0.67; 95% CI 0.53- 0.85; P < 0.001) for mortality as compared with the no-statin group (n = 562). No significant reduction in rate of VT/VF.
Tavazzi *et al.* 2008 (GISSI-HF trial) ^13^	RCT	4,574	Rosuvastatin	3.9 years	All-cause mortality, cardiovascular hospitalizations	No significant reduction in mortality (adjusted HR 1.00; 95.5% CI 0.898- 1.122; p=0.943) and cardiovascular hospitalization (adjusted HR 1.01; 99% CI 0.908-1.112; p=0.903).
Alehagen *et al.* 2015 ^22^	Prospective cohort	21,864	N/A	1 years	All-cause mortality	Reduction in all-cause mortality (HR 0.81; 95% CI 0.76-0.86; P<0.001)

Despite the above results, certain concerns regarding the generalizability of the trial results to the entire HFrEF population have been raised. The study population in both trials had a mean age of 73 years and most of the participants were already in advanced HF stages, namely New York Heart Association classification III and IV ^14, 65^. The observation from the CORONA trial that the patients in the lowest NT-proBNP tertile had a significantly lower risk of the primary adverse outcomes suggested that patients with less severe HF might benefit from statin therapy ^57^. Another study also found that the benefits of simvastatin toward reduction in major vascular events was relatively smaller in patients with higher NT-proBNP level ^66^. Moreover, the results of the GISSI-trial might have been impacted by compliance issues, as about one third of the study participants discontinued therapy for various reasons ^13^. Furthermore, the CORONA and GISSI-HF trials also assessed rosuvastatin at a low dose, and therefore the results cannot be inferred to a higher dose or different types of statins. It is worth recalling that rosuvastatin is a hydrophilic statin, which has poor uptake by cardiac muscles ^12^. On the other hand, lipophilic statins (e.g. atorvastatin, simvastatin, pitavastatin), which have better penetration into cardiac muscle cells and therefore possibly better influence the myocardium through pleiotropic effects ^12, 40, 67^, leading to the improvement in outcomes of patients with HF ^20, 43, 44, 68, 69^. In addition, other small randomized studies have shown that statins can improve certain surrogate endpoints in HF (e.g. LVEF, BNP, inflammatory markers), but not major outcomes (e.g. mortality, cardiovascular hospitalization), although it appears that most of the studies might not be adequately powered to assess major clinical outcomes ^12, 30, 70– 77^.

A more recent, albeit observational, nationwide prospective study, propensity score matched 21,864 patients with HFrEF from 86% of all Swedish hospitals. Statins were associated with reduced all-cause mortality (HR 0.81; 95% CI 0.76-0.686; p < 0.001), cardiovascular mortality, cardiovascular hospitalization, combined all-cause mortality, and HF hospitalization ^22^. The results concur with other observational studies that were reported both before and after the publications of CORONA and GISSI-HF trials ^15, 59, 62, 78– 80^. In addition, a recent meta-analysis ^20^ including 88,100 patients from 17 studies (2 randomized controlled trials and 15 cohorts) found that statin therapy was associated with reduced all-cause mortality and other secondary endpoints in patients with HF, similarly to other observational studies. The benefit was also consistent in HFrEF patients in the subgroup analysis ^20^. It has been suggested that the reason for the different results between large randomized trials and real-life cohort studies is that the randomized cohorts might not be representative of those in clinical practice as well as the dose and type of statins chosen in randomized trials, among other reasons ^15, 22, 25^. Therefore, additional randomized trials using statins other than rosuvastatin with more generalized inclusion in HFrEF populations may be warranted.

## Statins in heart failure with preserved ejection fraction: current evidence

Unlike HFrEF, no large RCTs have been conducted to assess the benefits of statins in patients with HFpEF. However, many studies have suggested the benefits of statins in this population ^20, 21, 39, 81– 86^. A list of relevant clinical studies of statins in patients with HFpEF is presented in Table 3. In a preliminary report of 137 consecutive patients with HF with LVEF ≥ 50% where half of the patients received statins, statin therapy was associated with lower mortality (RR 0.22; 95% CI 0.07-0.64; p = 0.006) ^82^. An observational prospective study following 9,140 patients with HFpEF in the Swedish Heart Failure Registry found that statin therapy was associated with decreased 1-year all-cause mortality (HR, 0.80; 95% CI: 0.72-0.89; P < 0.001), as well as decreased cardiovascular and composite all-cause mortality or cardiovascular hospitalization ^81^. Another multicenter prospective observational study in Japan with 3,124 patients with HFpEF also showed a similar decrease in mortality in patients with statins at three years (adjusted HR 0.74; 95% CI; 0.58-0.94; p < 0.001) ^83^. In the United States, a retrospective cohort study of 13,440 patients with HF, of which 7,563 had HFpEF (LVEF ≥ 50%) showed that statin use was associated with decreased mortality (HR 0.73; 95% CI 0.66-0.81; p < 0.001) in patients with HFpEF but not in patients with HFrEF or mid-range ejection fraction (HFmrEF) ^84^. Several meta-analyses have been conducted to assess the effects of statins on mortality in patients with HFpEF and have suggested similar findings of reduced mortality rate ^20, 39, 85, 86^.

**Table 3.** Relevant clinical studies with the primary objective of determining the effects of statins in patients with heart failure with preserved ejection fraction.

Study, year	Study design	N	Type of statin	Follow up time	Primary endpoints	Results
Fukuta *et al.* 2005 ^82^	Prospective cohort	137	Atorvastatin, simvastatin, pravastatin	21 months	All-cause mortality	Reduction in all-cause mortality (adjusted RR 0.20; 95% CI: 0.06 to 0.62; P=0.005)
Roik *et al.* 2008 ^91^	Prospective cohort	146	Simvastatin, atorvastatin, lovastatin, 2	12 months	All-cause mortality and cardiovascular hospitalization	Reduction in all-cause mortality (HR 0.24; 95% CI:0.07 - 0.90; P < 0.05) and cardiovascular rehospitalization rate (HR = 0.55; 95% CI: 0.33 - 0.92; P< 0.05)
Shah *et al.* 2008 ^92^	Retrospective cohort	13,533	N/A	3 years	All-cause mortality	Reduction in all-cause mortality (adjusted RR 0.73; 95% CI: 0.68– 0.79)
Liu *et al.* 2014 ^86^	Meta-analysis	17,985	N/A	N/A	All-cause mortality	Reduction in all-cause mortality (RR 0.60; 95% CI 0.49 to 0.74; p <0.001).
Tehrani *et al.* 2010 ^93^	Retrospective cohort	270	N/A	5 years	All-cause mortality and hospitalization	Reduction in all-cause mortality (HR 0.65; 95% CI: 0.45–0.95; P = .029). No significant difference in hospitalization rate
Nochioka *et al.* 2015 ^83^	Prospective cohort	3,124	N/A	36 months	All-cause mortality and hospitalization for worsening heart failure	Reduction in all-cause mortality (HR 0.71; 95% CI: 0.62-0.82; P < 0.001.) No significant reduction in hospitalization for worsening heart failure.
Alehagen *et al.* 2015 ^81^	Prospective cohort	9,140	N/A	12 months	All-cause mortality	Reduction in all-cause mortality (HR 0.80; 95% CI 0.72–0.89; P<0.001)
Fukuta *et al.* 2016 ^85^	Meta-analysis	5,536	N/A	N/A	All-cause mortality	Reduction in all-cause mortality (OR 0.69; 95% CI 0.49–097; P=0.030)
Lee *et al.* 2018 ^84^	Retrospective cohort	7,563	N/A	30 months	All-cause mortality	Reduction in all-cause mortality (HR 0.73; 95% CI 0.66-0.81; p < 0.001)
Tsujimoto *et al.* 2018 ^94^	Data derived from TOPCAT trial	3,378	N/A	3.3 years	All-cause mortality	Reduction in all-cause mortality (HR 0.79; 95% CI 0.63-0.99; P=0.04)
Marume *et al.* 2019 ^21^	Prospective cohort	414	N/A	25 months	All-cause mortality	Reduction in all-cause mortality (HR 0.21; 95% CI 0.06–0.72; P=0.014)

The studies mentioned above had included patients with coronary artery disease. In a most recent multicenter observational prospective cohort study in Japan evaluating 414 HFpEF patients without coronary artery disease, the use of statins was associated with a substantial decrease in 3-year mortality both in the entire and propensity score-matched cohorts ^21^. Moreover, the subgroup analyses also showed consistent benefits of statins across all range of blood cholesterol and HF severity ^21^. This study further suggested that statins might be beneficial for HFpEF patients even in those without coronary artery disease. Interestingly, it has been suggested that the findings where benefits of statins were consistently observed in HFpEF whereas in HFrEF the results were mixed might be due to greater effects of pleiotropic properties of statins on the pathophysiology of HFpEF ^87, 88^ and patients’ comorbidities ^83, 88^. While these findings appear promising, it is important to emphasize that these observational studies are only hypothesis-generating. Well-designed RCTs are needed to confirm the effects of statin therapy in HFpEF patients.

**Figure 1.**
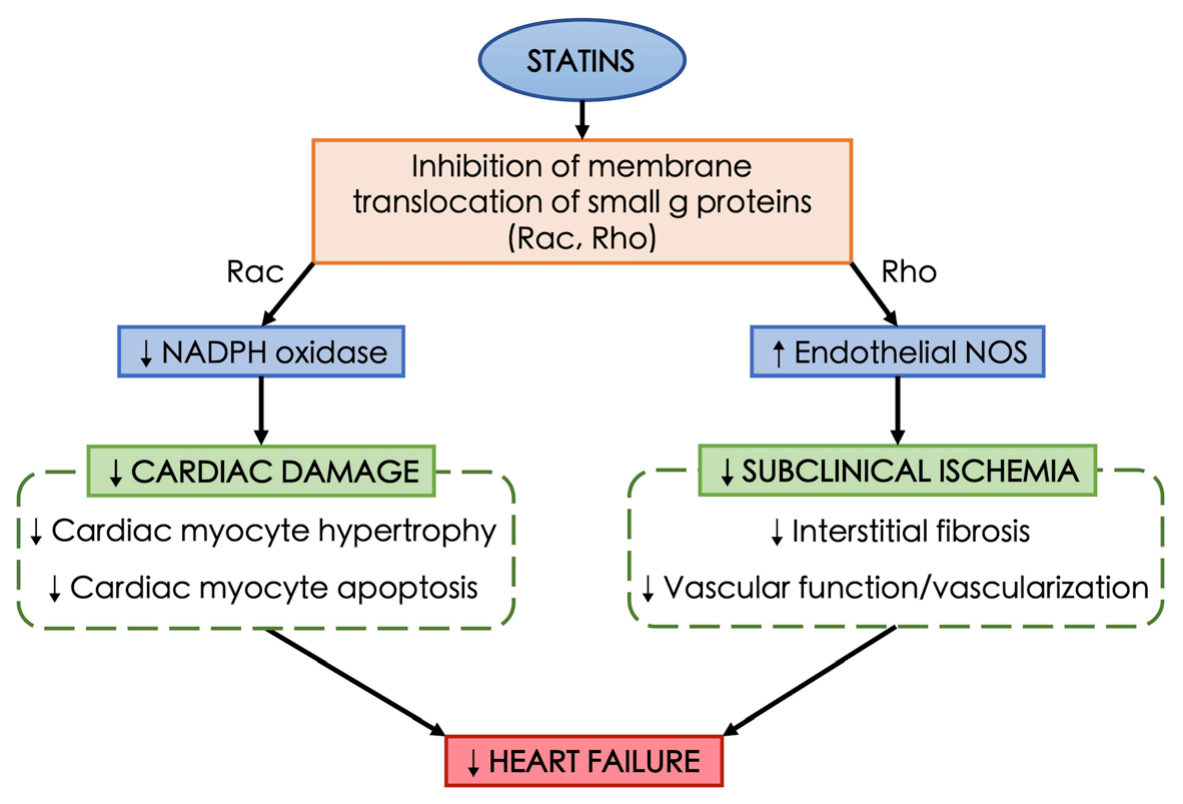
Proposed mechanism of pleiotropic effects of statins and heart failure prevention. Statins causes inhibition of membrane translocation of small g proteins, such as Rac and Rho. Inhibition of Rac pathway leads to the reduction of NADPH oxidase which results in less cardiac damage including cardiac myocyte hypertrophy and apoptosis. Similarly, inhibition of Rho pathway causes increased endothelial nitric oxide synthase (NOS) production leading to lower subclinical ischemia by reducing interstitial fibrosis, vascular function, and vascularization. Both mechanisms are proposed to prevent heart failure.

**Figure 2.**
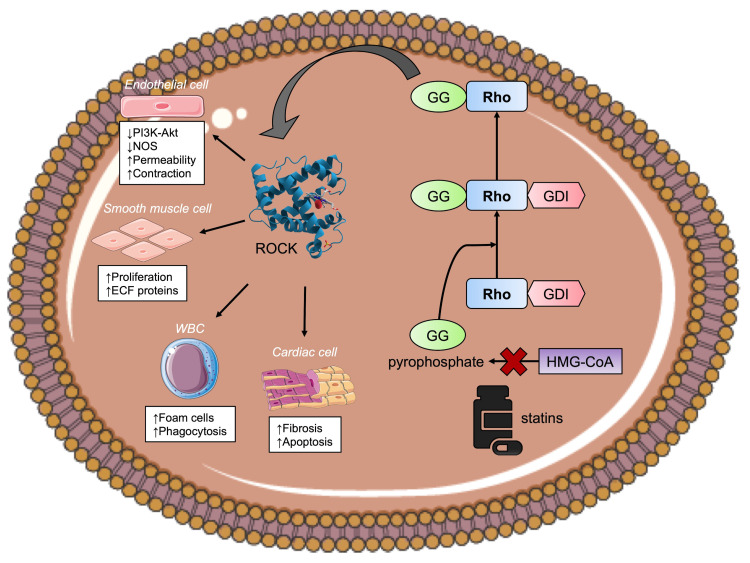
The mechanism of statins and regulation of the Rho GTPase cycle. Without statins, HMG-CoA is converted to mevalonate and subsequently geranylgeranyl (GG) pyrophosphate. The GG component is added to a complex of Rho protein and guanine nucleotide dissociation inhibitors (GDI) and therefore activates the Rho kinase (ROCK) pathway. ROCK mediates the downstream effects of Rho and has effect on endothelial cells, white blood cells, smooth muscle cells, and cardiac myocytes. The effect of statins inhibits the conversion of HMG-CoA to GG pyrophosphate which results in downregulation of ROCK and its associated effects on different target cells.

Possible unique pleiotropic effects of statins in patients with HFpEF have not been clearly defined. Statins may help prevent left ventricular hypertrophy and fibrosis in HFpEF ^21, 89, 90^. Experimental studies have shown that statins can improve diastolic dysfunction ^95, 96^ and attenuated left ventricle stiffness ^97^, both of which are important underlying pathogeneses of HFpEF. Anti-inflammatory and anti-oxidant effects of statins may be beneficial in HFpEF as systemic inflammatory state plays a role in the pathogenesis of HFpEF ^87^. The pleiotropic effects of statins are illustrated in Figure 1. Pathogenesis of HFpEF may also be driven by several comorbidities such as overweight/obesity, diabetes mellitus, renal dysfunction, and hypertension ^87^, as current evidence suggests that statins have been shown to be beneficial in these conditions as well ^98– 100^. Lipophilic statins may have a favorable effect on sympathetic activity in HFpEF, as increased sympathetic activity is associated with the pathogenesis of HFpEF ^101^. Further mechanistic studies focusing on the underlying cellular and molecular changes in HFpEF caused by statins are needed. Possible roles of collagen synthesis, matrix metalloproteinases inhibition, Rho kinase 1 gene expression in relation to diastolic dysfunction in HFpEF may be an area of further studies ^96^. The mechanism of stains and regulation of the Rho GTPase cycle is illustrated in Figure 2.

## Other lipid lowering medications and their role in heart failure

Proprotein convertase subtilisin–kexin type 9 inhibitors (PCSK9i) have not been shown to reduce death or hospitalizations from worsening HF in two large RCTs ^102, 103^. Icosapent ethyl, a form of highly purified omega-3 eicosapentaenoic acid ethyl ester (EPA), is used to lower triglyceride levels. EPA has been shown to effectively reduce ischemic events and cardiovascular death among patients who have high triglyceride levels and are already on statin therapy ^104^. EPA may also reduce new HF hospitalization if a high level of on-treatment EPA in the blood is achieved ^105^.

## Conclusions and future perspectives

The current evidence we have for statins in the treatment of established HF is far from satisfactory. Our review shows contradicting evidence for statin therapy in HFrEF. Despite negative results from two large RCTs, many real word data do suggest benefits of statins in HFrEF. Thus, an adequately powered randomized placebo-controlled trial to determine the effects of statins other than rosuvastatin with more generalized inclusion criteria, preferably with long term follow up, is needed to clarify the benefits of statins in HFrEF. On the other hand, the benefits of statins in improving clinical outcomes among HFpEF patients were consistently reported by observational studies both in the United States and international institutions, even in patients without coronary artery disease. Nevertheless, there exists a dire need for new treatment options for patients with HFpEF as the current choice of pharmacological treatments is very limited. A future RCT comparing the treatment outcomes of statin therapy in HFpEF is also needed and would contribute a significant impact in closing the knowledge gap of HF management. Moreover, a direct head-to-head comparison of hydrophilic versus lipophilic statins in HF will also help elucidate the hypothesis that lipophilic statins might be more appropriate for patients with HF. To generate this much needed evidence, artificial intelligence - a rapidly developing field in medicine - has the potential to be incorporated in the design and execution of future RCTs ^106^. Application of artificial intelligence may improve efficacy of RCTs by improving the patient’s selection process, minimizing measurement errors when assessing endpoints, or even providing trials with synthetic control groups ^107^.

More recently, network medicine is a novel discipline that studies and integrates heterogenous interconnected molecular and genetic data as a network and identifies perturbations in these networks that ultimately causes disease ^108, 109^. This concept has been applied in cardiovascular medicine and may also provide the insights into spatio-temporal statin-mediated mechanisms of statins in patients with HF. For example, a group of investigators has conducted a network analysis integrating myocardial infarction drugs, drugs interactors, drug targets, and myocardial infarction disease genes onto the human interactome ^110^. By integrating gene-disease associations, they were able to identify “drug-target-disease modules”, which provide a better understanding of the drug actions and mechanisms ^110^. This approach should also be pursued to elucidate the mechanisms of statins in patients with HF in addition to the previously existing clinical evidence from RCTs.

## Data availability

All data underlying the results are available as part of the article and no additional source data are required.
