# Supplementary material for: Therapeutic implications of statins in heart failure with reduced ejection fraction and heart failure with preserved ejection fraction: a review of current literature
Source: F1000Res. 2021 Jan 12;10:16. [Version 1] doi: 10.12688/f1000research.28254.1 (PMC9982192; doi:10.12688/f1000research.28254.1)
Supplement: Peer review history

# Peer review: Reviewer response for version 1

**DOI:** 10.5256/f1000research.31250.r164582

**Published:** 2023-03-02

**Authors:** Rui Valdiviesso

**Affiliations:** 1 Faculdade de Ciências da Nutrição e Alimentação da Universidade do Porto, Porto, Portugal; 2 ProNutri, CINTESIS@RISE, Porto, Portugal

This manuscript presents a much needed review of the existing literature regarding the effects of statins on clinical outcomes of HF patients with preserved or reduced ejection fraction. The article is clear, informative, and correctly organised. It cites the most relevant clinical studies in the field, such as the CORONA and GISSI-HF, while giving relevant context on the strengths and limitations of each study. The important and many times overlooked behaviour of lipophilic vs. hydrophilic statins was also included in this review.

 Although the discussion could go slightly deeper, particularly regarding other outcomes that could add to the utility of statin therapy in HF patients, the conclusions seem correct and in adequacy with the findings of the reviewed evidence. 

 Other impression that I feel obliged to add to this review is that these findings seem to be in line with my own impressions from both clinical practice with HF patients and studies I conducted or participated in. 

 The article is also very informative and written in a pedagogical fashion. Figures 1 and 2 were tastefully added and are useful illustrations to the implied mechanisms. 

 In face of my review, I recommend the indexing of this manuscript. 

 My congratulations to the authors.

Is the review written in accessible language?

Yes

Are all factual statements correct and adequately supported by citations?

Yes

Are the conclusions drawn appropriate in the context of the current research literature?

Yes

Is the topic of the review discussed comprehensively in the context of the current literature?

Yes

Reviewer Expertise:

Heart failure; frailty; sarcopenia; nutritional and functional status in heart failure; muscle strength; medication affecting muscle function in heart failure.

I confirm that I have read this submission and believe that I have an appropriate level of expertise to confirm that it is of an acceptable scientific standard.

# Peer review: Reviewer response for version 1

**DOI:** 10.5256/f1000research.31250.r87223

**Published:** 2021-06-14

**Authors:** Claudio Bilato

**Affiliations:** 1 Division of Cardiology, West Vicenza General Hospitals, Vicenza, Italy

This is a comprehensive review on the role of statins in heart failure both with reduced and preserved ejection fraction. The paper is well-written, informative and easy to read. The authors reported the most important studies and the meta-analysis on the topic in a very exhaustive manner.

 Although some data are not analyzed in-depth the manuscript deserves indexing because it is very educational and useful.

Is the review written in accessible language?

Yes

Are all factual statements correct and adequately supported by citations?

Yes

Are the conclusions drawn appropriate in the context of the current research literature?

Yes

Is the topic of the review discussed comprehensively in the context of the current literature?

Yes

Reviewer Expertise:

Clinical Cardiology

I confirm that I have read this submission and believe that I have an appropriate level of expertise to confirm that it is of an acceptable scientific standard.

# Response

**Published:** 2021-06-14

**Authors:** Panupong Hansrivijit

**Affiliations:** UPMC Pinnacle, USA

Thank you for your time and comment.
